# Extract of the Suprarenal Gland in Dentistry

**Published:** 1902-12

**Authors:** William H. Potter


### Reviews of Dental Literature.

Extract of the Suprarenal Gland in Dentistry. By Fritz Moeller, Berlin.[1]

Extract of the suprarenal gland has been used for the last ten years, especially in England and America. Owing to the variations in the preparation of the product its use has been much limited. During the last year Professor Rosenberg, in Berlin, has undertaken important experiments with this agent. According to him, the anæmia which follows the application of the extract to the mucous membrane of the nose is responsible for the lowering of the sensitiveness of this membrane. When combined with cocaine it produces an anæsthesia which penetrates even to the bones. The use of the drug is without danger, and does not induce a drug habit. Rosenberg has succeeded by the use of the extract in amputating the middle turbinate bone without loss of blood to the patient and without pain. In speaking of the physiological action of the suprarenal gland, the author says that it constantly secretes a substance which increases the blood-pressure and holds it at a certain level. Its presence is necessary for life. In the case of animals where it has experimentally been removed, death has followed in every case.

[1] Nebennierenextract in der Zahnheilkunde, von Zahnarzt Fritz Moeller, in Berlin. Deutsche Monatsschrift für Zahnheilkunde, 23 September, 1902.

The bodily temperature which is lowered by injury to the gland can be raised by an injection of the suprarenal extract.

The chemical properties of the extract are not destroyed by heat until a temperature of 110° C. is reached, so that it can readily be sterilized. When treated with ferric chloride it takes on a characteristic green color, which, by absorption of oxygen from the air, becomes red and later brown. Locally applied, it increases the blood-pressure and develops a local anæmia.

Experiments upon dogs, rabbits, and cats show that when twenty grammes of the five per cent. solution is injected for eight days in succession they still hold their weight and eat well. The author has injected into his own lower arm eight cubic centimetres of a five per cent. solution in one day. No elevation of the pulse or temperature could be determined. After twenty-four hours sugar appeared in the urine, but disappeared in twelve hours' time. In experiments upon animals, Bluhn and Zülzer have established the fact that after an injection of the extract sugar appears in the urine, but no impairment of the health follows.

The author considers the American and English preparations of suprarenal capsule unsuited for dental use. He says that in order to make them stable they are mixed with bromide of camphor compounds or iron salts. These substances influence unfavorably the hæmostatic action of the preparation. The author speaks favorably of the preparation made by Freund & Redlich, Berlin. This preparation is, however, not altogether satisfactory to him, and he makes one for himself as follows: From fresh calves' and cows' suprarenal glands a one per cent. solution is made; this is immediately sterilized and sealed up in brown glass capsules holding five cubic centimetres. This preparation is almost as clear as water, and free from albumin, lecithin, and pyrocatechin, and is of remarkable efficiency. After the capsules are cold they are again heated in a sand-bath at 108° C. for twenty-four hours. The author then prepares capsules which contain the following drugs: Cocaine mur., 0.01; morph. mur., 0.001; aqua destill., 1.00. As preparatory to use the contents of the two capsules are mixed. The injection is made, for purposes of extraction, one to two millimetres from the edge of the mucous membrane under the periosteum, in a direction parallel to the root of the tooth, and the whole region is rendered anæmic. Freienstein's infiltration syringe is used. The author uses the preparation freely, sometimes five cubic centimetres. The preparation contains less cocaine than the weakest Schleich solution. The extraction is entirely painless and the bleeding astonishingly small.

WILLIAM H. POTTER.

